# Retraction: One-pot odourless synthesis of thioesters via in situ generation of thiobenzoic acids using benzoic anhydrides and thiourea

**DOI:** 10.3762/bjoc.19.85

**Published:** 2023-08-07

**Authors:** Mohammad Abbasi, Reza Khalifeh

**Affiliations:** 1 Chemistry Department, Faculty of Sciences, Persian Gulf University, Bushehr 75169, Iran; 2 Department of Chemistry, Shiraz University of Technology, Shiraz, Iran

**Keywords:** benzoic anhydride, Michael addition, nucleophilic displacement, thioester, thiourea

This article was retracted due to the improper manipulation of NMR spectra with the help of software to obscure peaks corresponding to impurities and solvent.

